# Analysis of Selected Properties of Injection Moulded Sustainable Biocomposites from Poly(butylene succinate) and Wheat Bran

**DOI:** 10.3390/ma14227049

**Published:** 2021-11-20

**Authors:** Emil Sasimowski, Łukasz Majewski, Marta Grochowicz

**Affiliations:** 1 Department of Technology and Polymer Processing, Faculty of Mechanical Engineering, Lublin University of Technology, 20-618 Lublin, Poland; e.sasimowski@pollub.pl; 2 Department of Polymer Chemistry, Institute of Chemical Sciences, Faculty of Chemistry, Maria Curie-Sklodowska University, 20-400 Lublin, Poland; mgrochowicz@umcs.pl

**Keywords:** composite, injection moulding, biofiller, bioplastic, thermal properties, thermo-mechanical properties, mechanical properties, agro-waste materials, agro-flour filler

## Abstract

The paper presents a procedure of the manufacturing and complex analysis of the properties of injection mouldings made of polymeric composites based on the poly(butylene succinate) (PBS) matrix with the addition of a natural filler in the form of wheat bran (WB). The scope of the research included measurements of processing shrinkage and density, analysis of the chemical structure, measurements of the thermal and thermo-mechanical properties (Differential Scanning Calorimetry (DSC) and Thermogravimetric Analysis (TG), Heat Deflection Temperature (HDT), and Vicat Softening Temperature (VST)), and measurements of the mechanical properties (hardness, impact strength, and static tensile test). The measurements were performed using design of experiment (DOE) methods, which made it possible to determine the investigated relationships in the form of polynomials and response surfaces. The mass content of the filler and the extruder screw speed during the production of the biocomposite granulate, which was used for the injection moulding of the test samples, constituted the variable factors adopted in the DOE. The study showed significant differences in the processing, thermal, and mechanical properties studied for individual systems of the DOE.

## 1. Introduction

Over the past several years, environmental issues have been increasingly raised, prompted by alarming reports of the environmental pollution caused by excessive use of petrochemical plastics [1,2,3,4,5,6]. One of the rapidly developing ways of prevention of the increasing pollution is the development and widespread use of biocomposites with natural fillers. In particular, the biocomposites are based on biodegradable or compostable polymers that are derived from natural sources or synthesized from substrates of natural origin [7,8]. Numerous complex compositions of multiple biodegradable polymers in various ratios are also used for that purpose [9,10,11,12]. Examples of such polymeric materials used to produce biocomposites include polylactide [13], polyvinyl alcohol [14], poly(hydroxyalkanoates) [15], polycaprolactone [16], and one of the more interesting—poly(butylene succinate) (PBS) [17]. PBS has very good functional properties that allow it to be widely used even in specific applications [18,19]. It is also characterized by very good mechanical and processing properties, which could classify this polymer as a structural material of common use [20,21,22]. However, PBS, like most of biodegradable polymers, has one significant disadvantage—a manifold higher price compared to traditional polyolefins of petrochemical origin, such as polypropylene or polyethylene [19,23,24]. This reduces its industrial popularity by excluding it from common use and marginalizing it to industries with high production costs [25,26]. The reason for the high costs consists mainly in the complex process of preparation and the high price of substrates but also the necessity of their drying, storage, and transportation in special conditions [27,28,29,30,31]. Therefore, the area of our current interest includes PBS-based polymer biocomposites with the addition of low-cost natural fillers, whose addition facilitates the possibility to reach the price competitiveness level, but often also provides a unique set of properties [21,32,33].

The literature includes numerous papers dealing with the manufacturing and properties of biocomposites on a PBS matrix with the addition of various natural fillers. Examples of such fillers are shredded wood shavings [34]; ground bran of cereals (wheat [35] and rice [36]); nut shells (pistachios [37], peanuts [38], and coconut [39]); and seeds (almonds [40]) but also dried pomace (apple [41] and grape [42]) or even wine lees [43]. The composition of all natural fillers of plant origin is based mainly on cellulose, hemicellulose, and lignin, but they differ in structure as well as in the proportion of their main components and the content of additional substances, such as simple and complex sugars, proteins, fats, and water [44,45,46]. Due to these differences, each lignocellulosic filler (LCF) will modify the properties of the polymer biocomposite in its own individual way. However, it can be generally assumed that the addition of the LCF positively affects the degradation rate and improves the stiffness of the composites [42,47,48] but also reduces the density and wear of the processing machine components compared to the mineral fillers [49,50,51]. However, the use of powdered byproducts of natural origin as fillers has some disadvantages and entails technological issues. Firstly, there is a decrease in the processability of composites due to the content of a significant amount of moisture and the increase of their viscosity and resistance during processing [52,53,54,55]. It is associated with an increased force on the drive system of the processing machine, a decrease in process efficiency and a risk of pore formation and hydrolysis during processing [52,53,56,57]. Secondly, the presence of LCF reduces the thermal resistance of the composites due to the low thermal decomposition temperatures of their structural components, which can be as high as approx. 150 °C. Therefore, PBS is suitable for the production of biocomposites with natural fillers because it has a low melting point (about 115 °C) [17,58,59,60,61]. Thirdly, the mechanical strength of LCF biocomposites is usually inversely proportional to the filler content [4,21,50,62]. The decrease in strength is usually related to the strength of the interfacial interactions at the polymer matrix/filler boundary. Due to their chemical structure, LCFs are hydrophilic in nature, whereas long polymer chains are hydrophobic or moderately hydrophilic due to the presence of local functional groups capable of forming hydrogen bonds [63,64]. Many authors indicate a significant decrease in the tensile strength of PBS biocomposites with the addition of powdered natural fillers. The decrease in strength is often higher the greater the filler content and can reach values of up to 50% [18,21,36,38,40,41,42]. The reduction in strength of biocomposites relative to unfilled polymeric materials is therefore an inherent aspect of the use of natural fillers. PBS, on the other hand, is hydrophilic in nature, and its water wetting angle is 70° [65] so that the level of interaction of PBS with the filler remains at a satisfactory level. This, combined with the good strength of neat PBS, makes it possible to efficiently produce biocomposites even with a high filling degree while maintaining satisfactory values of mechanical resistance parameters [66]. Nevertheless, many authors decided to use a compatibilizer during the manufacturing of the biocomposites, based on a PBS matrix with an addition of natural fillers. Due to the low popularity of PBS, there are no commercially available compatibilizers based on PBS, as is the case for polyethylene, where polyethylene grafted with maleic anhydride is widely available. In the case of PBS, maleinized, or epoxidized vegetable oils [38,40,67] or coupling agents based on, e.g., silanes [48,68,69] are used as compatibilizers in scientific research. Modified vegetable oils are most often ineffective and even cause a reduction in tensile strength, stiffness, and impact strength with respect to the PBS with filler but without oils [38,40,67]. Organosilane-based compatibilizers can improve the mechanical properties relative to biocomposites without a compatibilizer, but their cost is significant, and they exhibit significantly higher efficiencies over fibrous fillers than powdered ones [48,69]. Some authors choose to produce maleic anhydride-grafted PBS under laboratory conditions, such as by extrusion of reactive PBS and maleic anhydride in the presence of dicumyl peroxide. Despite the high efficiency of this compatibilizer, its use drastically increases the cost of manufacturing PBS matrix composites with natural filler [70,71,72]. The main purpose of using natural-waste fillers, which are most often technological waste from food or agricultural industry, is to reduce the cost of expensive polymeric materials such as PBS [49,73]. Therefore, it should be noted that the production of a compatibilizer or the use of a commercially available one significantly increases the cost of manufacturing the whole biocomposite, and the obtained strengthening effects are moderate or unsatisfactory. Thus, the use of compatibilizers during the manufacturing of PBS-based biocomposites is technically as well as economically unjustified [25,32,74].

The scientific literature abounds in papers dealing with the subject of biocomposites made of a PBS matrix with the addition of various fillers of natural origin, including fillers made of agricultural and food industry wastes. Despite the above, there is a shortage of works describing in detail the manufacturing process and characterizing the properties of compositions made of poly(butylene succinate) with the addition of ground wheat bran, which is a technological waste in the production of white flour. In the following work, an extensive and detailed analysis of selected properties of PBS injection mouldings filled with crushed wheat bran was carried out. The aim of this study was to evaluate the influence of wheat bran content and extruder screw speed during the extrusion of biocomposite pellets on the properties of the injection-moulded parts produced from them. The characteristics of the changes in the processing and the physical, structural, thermal, thermo-mechanical, and mechanical properties were determined as functions of variable factors; this was followed by an extensive analysis of the obtained results.

## 2. Experimental

### 2.1. Test Stand

Injection moulding of the biocomposite was carried out using an Arburg Allrounder 320C (Arburg, Lossburg, Germany) screw injection moulding machine equipped with a dual cavity mould to produce specimens for strength testing. The shape and dimensions of the samples were in accordance with ISO 294-1:2017-07 [75]. The specimens were dog-bone-shaped with a total length of 150 mm and a thickness of 4 mm; the width of the measuring part was 10 mm, and the grip part was 20 mm. Due to the danger of the thermal decomposition of the biocomposite components, low temperatures were applied during processing. The temperature of the plasticizing system was 30 °C in the feed zone, and in the individual heating zones it was: I–125 °C, II–145 °C, III–155 °C, and IV–160 °C; the injection nozzle temperature was 155 °C. The temperature of the thermostated mould was 25 °C. The injection of the biocomposition was performed at the following settings: maximum injection pressure 120 MPa, polymer flow rate 20 cm^3^/s, packing pressure 110–80 MPa, packing time 15 s, and cooling time 20 s. In the case of the highest bran fraction of 50% (DOE layout 8), the injection pressure was increased by 130 MPa and the packing pressure to 120–80 MPa, which made it possible to eliminate the incomplete filling of mould cavities occurring at the lower values of these parameters.

### 2.2. Materials

The components of the studied biocomposition are: PBS constituting its matrix and filler in the form of wheat bran. A PBS designed for general-purpose injection moulding, trade name BioPBS FZ91 PB [76], was used to produce the biocomposition samples. This material is produced from bio-based succinic acid and 1,4-butanediol by PTT MCC BIOCHEM CO., Ltd., Bangkok, Thailand. The wheat grain husks, or wheat bran (WB), used in the biocomposition came from a local mill near Lublin (Poland). They are a waste product from the refining of white flour. The bran takes the form of thin flakes several millimetres thick, composed of fibrous substances, such as cellulose, lignin, and hemicellulose. They also contain phytic acid, oligosaccharides, and non-starch polysaccharides, as well as fats and proteins, in their composition [77,78].

### 2.3. Research Programme and Methodology

Experimental tests were carried out according to the adopted DOE: central, composite, rotatable with star point distance = 1.414. The following independent variables—adjustable conditions of the process—were assumed: mass content of wheat bran introduced into poly(butylene succinate) *u* = 10 ÷ 50%wt and extruder screw speed *n* = 50 ÷ 200 min^−1^ when obtaining the processed biocomposite pellets. A detailed analysis of the extrusion process and properties of the compositions obtained were presented in a previous paper [66]. The experimental design and test results obtained—the mean values of the dependent variables studied—are presented in Table 1 and Table 2. Measurements were made in at least five replicates.

The following dependent (observed) variables were adopted in the experimental study: processing longitudinal *S_L_*, transverse *S_T_* and perpendicular *S_P_* shrinkage [%], density *ρ* [g/cm^3^], heat deflection temperature HDT [°C], Vicat softening temperature VST [°C], hardness *H* [MPa], impact strength [kJ/m^2^], tensile strength *σ* [MPa], Young’s modulus *E* [MPa], and elongation at break *ε* [%]. The measurements carried out according to the adopted design of the experiment made it possible to approximate the relationship between the mentioned dependent and independent variables by means of a polynomial value of many variables consisting of the following members: constant value, linear terms, quadratic terms, and a two-factor interaction term (Equation (1)) [66], where Y is the predicted response value (Y stands for *H*, *S_L_, S_T,_ S_P,_ HDT, VST,* *ρ*, impact strength, *σ*, *E*, and *ε*), a_0_ is a constant value, and a_x_ are the regression coefficients.

(1) $$Y\left(n\cdot u\right)=a_{0}+a_{1}n+a_{2}u+a_{3}n^{2}+a_{4}u^{2}+a_{12}nu$$

Experimental tests of the injection mouldings made of the polymer compositions were carried out:

- Measurement of longitudinal shrinkage *S_L_* and transverse shrinkage *S_T_* and perpendicular shrinkage *S_P_* of the samples with the use of a caliper as per ISO 294-4:2005 [79]. The measurement was made with an accuracy of 0.01 mm.
- Standard density was measured according to ISO 1183-1 A [80] using the immersion method. The mass of the samples in air and in water was measured. In order to obtain full soaking of the sample, the processed products were kept immersed in water for 24 h and then measured.
- FTIR analysis was performed using a TENSOR 27 FTIR spectrophotometer (Bruker, Billerica, MA, USA), with ATR (Attenuated Total Reflectance). The measurement was performed with a diamond crystal, recording 16 scans per spectrum in the range of 600–4000 cm^−1^ with a resolution of 4 cm^−1^.
- Differential scanning calorimetry (DSC) measurements of biocomposite injection mouldings were performed according to ISO 11357-1:2016 [81] using a NETZSCH (Günzbung, Germany) model 204 F1 Phoenix DSC scanning calorimeter. Processing of the test data was carried out using the NETZSCH Proteus software. Measurements were made under the following conditions: heating cycle (I) with a heating rate of 10 K/min in the temperature range of −150 °C–140 °C; cooling cycle at a rate of 10 K/min within a temperature range of 140 °C–150 °C; heating cycle (II) at a rate of 10 K/min within the temperature range of −150 °C–140 °C; mass of measuring samples about 10 mg; and aluminium crucibles with pierced lids. On the basis of DSC curves obtained, the following findings were determined: crystallinity degree *X_c_*, melting enthalpy Δ*H_m_*, melting temperature *T_m_*, crystallization temperature *T_c_*, and glass transition temperature *T_g_* of the investigated biocomposite samples. The adopted inflection point of the DSC curve in the glass transition region corresponded to the glass transition temperature. While determining the degree of crystallinity, the relation (Equation (2)):

(2) $$X_{c}=\left(\frac{\Delta H}{\left(1-u\right)\times \Delta H_{100\%}}\right)\times 100\%$$

The adopted Δ*H*_100%_ value for PBS in the calculation = 1103 J/g [82].

- Thermogravimetric (TG) measurements of the injection mouldings were carried out using a Jupiter STA 449 F1 thermal analyser (NETZSCH, Günzbung, Germany) in an oxidizing atmosphere. The gaseous products of the sample decomposition were analysed using an attached TENSOR 27 FTIR spectrophotometer from Bruker, (Germany). The measurements were carried out under the following conditions: temperature 40–800 °C, synthetic air flow rate 25 mL/min, sample mass about 12 mg, and measuring crucibles made of Al_2_O_3_.
- The heat deflection temperature (HDT) tests were performed using a Ceast HV3 apparatus manufactured by Instron (Turin, Italy) as per ISO 75-2:2013 [83]. Flat specimen alignment, B-measurement method (flexural stress 0.45 MPa), and a heating rate of 120 °C/h were used.
- Vicat softening temperature (VST) values were also determined on the Ceast HV3 apparatus by Instron (Turin, Italy) as per ISO 306:2013 [84]. The A120 measurement method was applied—10N force and 120 °C/h heating rate.
- The hardness was measured employing a ball indentation method with the use of an HPK 8411 hardness tester with a ball-shaped indenter of 5 ± 0.025 mm diameter. Measurements were made in accordance with ISO 2039-1:2004 [85]
- Unnotched Charpy impact tests were carried out in accordance with ISO 179-2:2020 [86] on a Type 639F impact hammer by Cometech Testing Machines (Taizhong, Taiwan). The pendulum used had a maximum energy of 5093 J. The samples for impact tests were made by cutting the measuring part out of injection-moulded, dog-bone-shaped samples, obtaining rectangular samples with dimensions of 80 × 10 × 4 mm.
- The strength properties, such as tensile strength σ [MPa], elongation at break ε [%], and Young’s modulus [MPa] were determined based on ISO 527-2 [87]. The tensile speed during the measurements was 50 mm/min. The measurements employed a Zwick Roell (Ulm, Germany) model Z010 testing machine.

## 3. Results

The collected results of the experimental investigations on the properties of the injection mouldings of poly(butylene succinate) (PBS) biocomposition filled with wheat bran are presented in Table 1 and Table 2. The collected experimental results were used to determine empirical models describing the influence of adjustable process conditions (independent variables) on the examined properties of biocomposition (dependent variables). The models were adjusted using the backward stepwise regression method. The applied Pareto chart of standardized effects allowed us to illustrate the influence of the members of the regression equations on the studied quantity (dependent variable). Statistically significant are the members for which the absolute values of the standardized effects exceed the vertical line corresponding to the assumed significance level *p* = 0.05.

### 3.1. Physical and Structural Properties

#### 3.1.1. Processing Shrinkage

Determined empirical models of the processing of longitudinal *S_L_*, transverse *S_T_*, and perpendicular *S_P_* shrinkage were presented by means of polynomials (Equations (3)–(5)):

(3) $$S_{L}=1.362225-0.016295u$$

(4) $$S_{T}=2.205955-0.020187u\;$$

(5) $$S_{P}=1.915118+0.031844u-0.001090u^{2}\;$$

**Figure 1:**
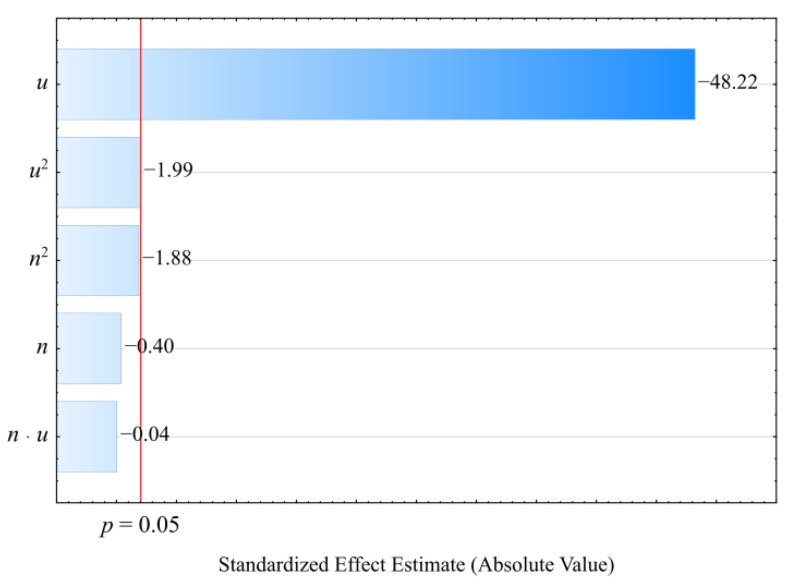
Pareto charts of the standardized effects for the empirical model of longitudinal shrinkage *S_L_*; the vertical line in the plot corresponds to the arbitrarily chosen level of significance (*p* = 0.05).

**Figure 2:**
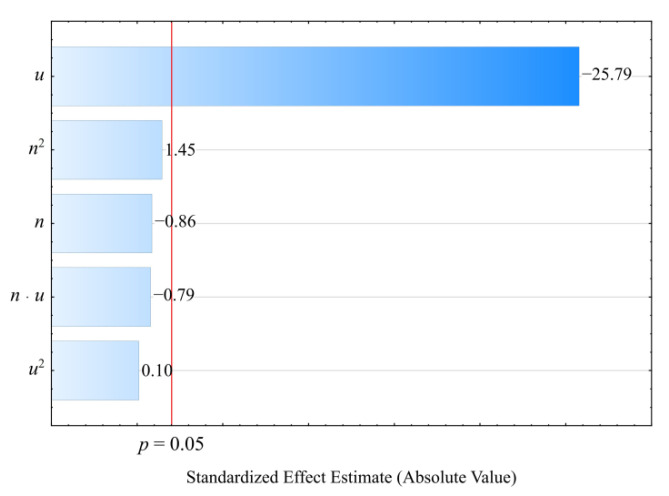
Pareto charts of the standardized effects for the empirical model of transverse shrinkage *S_T_*; the vertical line in the plot corresponds to the arbitrarily chosen level of significance (*p* = 0.05).

**Figure 3:**
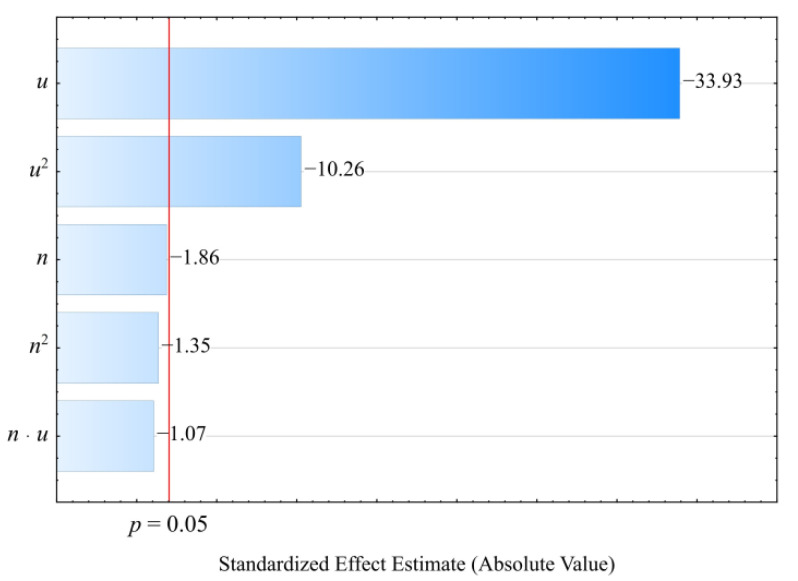
Pareto charts of the standardized effects for the empirical model of perpendicular shrinkage *S_P_*; the vertical line in the plot corresponds to the arbitrarily chosen level of significance (*p* = 0.05).

**Figure 4:**
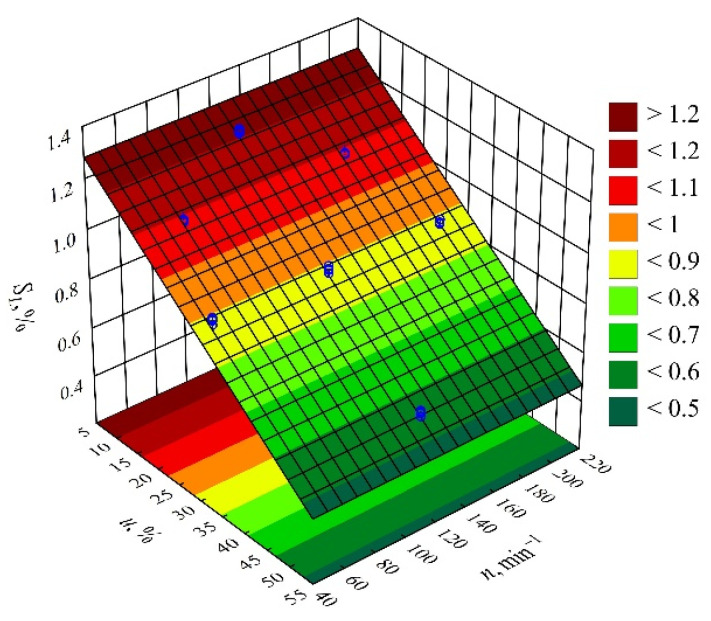
Response surface graph for the longitudinal shrinkage *S_L_* versus wheat bran content *u* and screw speed *n*.

**Figure 5:**
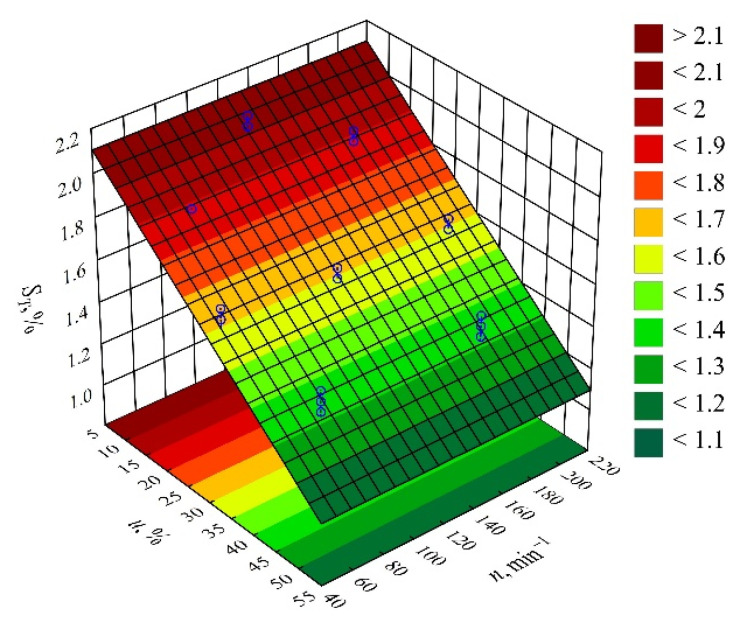
Response surface graph for the transverse shrinkage *S_T_* versus wheat bran content *u* and screw speed *n*.

**Figure 6:**
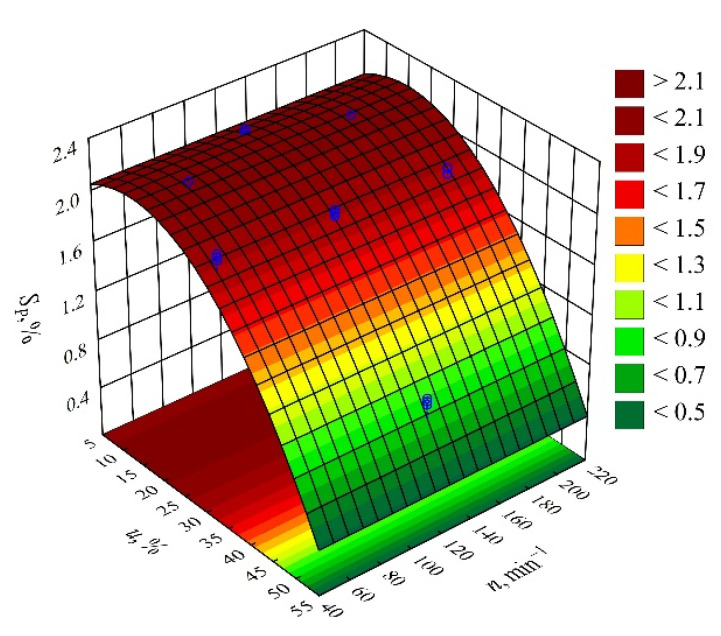
Response surface graph for the perpendicular shrinkage *S_P_* versus wheat bran content *u* and screw speed *n*.

The results of the statistical analyses of the adopted processing shrinkage models are presented in Table 3, Table 4 and Table 5. It was observed that the wheat bran content *u* has a significant effect on the types of processing shrinkage *S_L_*, *S_T_*, and *S_P_*, and that this relation is linear for the longitudinal and transverse shrinkage (Figure 1 and Figure 2). For perpendicular shrinkage *S_P_*, the quadratic term of the model with a negative effect is also statistically significant (Figure 3). Increasing the bran fraction results in a significant decrease in processing shrinkage (Figure 4, Figure 5 and Figure 6). The greatest reductions in shrinkage values obtained by increasing the wheat bran content *u* in the composition from 10 to 50% (DOE layouts 7 and 8) were 0.66% for longitudinal shrinkage *S_L_* (54% of the initial value), 0.92% for transverse shrinkage *S_T_* (45% of the initial value), and 1.21% for perpendicular shrinkage *S_P_* (57% of the initial value), respectively. No effect of the applied extruder screw speed *n* during the production of the polymeric composition and the occurrence of interactions between the variable factors studied on the processing shrinkage of the mouldings studied was observed.

In the case of mouldings made from the PBS alone, without the addition of bran, the processing shrinkage was higher and amounted to: longitudinal *S_L_* = 1.57 ± 0.007%, transverse *S_T_* = 2.40 ± 0.027%, and perpendicular *S_P_* = 2.22 ± 0.014%.

The processing shrinkage of injection-moulded parts made of partially crystalline polymers depends on many factors, which include heat transfer during cooling, volume shrinkage due to thermal expansion, flow-induced residual stresses, orientation of macromolecules, and crystallization. The above factors are in turn dependent on the processing parameters and properties of the injected material [88]. All of the measurement series studied are characterized by partial crystallinity [66], by which the effect of shrinkage anisotropy was observed. It is recognized that for partially crystalline materials, the highest shrinkage values are observed in the flow direction due to the flow-induced orientation of the macromolecules [89], while in the analysed case the measured values of the longitudinal processing shrinkage were found to be smaller than the values of the transverse and perpendicular shrinkage. This is related to the elastic recovery effect generated by the amorphous phase [90], whose share is significant and varies in the range of 27–46% depending on the filler share, as presented further in the description of the DSC studies. Moreover, the papers dealing with the shrinkage of semi-crystalline polymeric materials, including polyesters, demonstrated that when we take into account the additional factors affecting shrinkage, the processing shrinkage in this type of material occurs most intensively at the sample thickness [88,90]. This is consistent with the obtained shrinkage results for the tested PBS/WB biocomposites. The exceptions are the composites with the highest WB content (44% and 50%), for which the transverse shrinkage reached values higher than the perpendicular shrinkage. This is most likely to be due to a significant change in the material properties, such as viscosity and thermal conductivity, which leads to changes in cooling efficiency, the quality of pressure transmission in the flow system, and local flow rates. The value of shrinkage in the perpendicular direction occurring at the sample thickness is most susceptible to changes in the processing parameters during injection moulding [88,89,90].

The PBS/WB composites showed lower processing shrinkage values with respect to the unfilled PBS. This is due to the fact that the filler used is not subject to processing shrinkage. The effect of specific volume loss related to the crystallization effect during cooling is less pronounced with increasing filler content in the temperature and pressure ranges used during the injection moulding. This has been confirmed in previous work [66] via *p-v-T* tests.

#### 3.1.2. Density

The result of the performed modelling of the density *ρ* of the injection mouldings made of the compositions under study is an empirical model in the form of the polynomial (Equation (6)):

(6) $$\rho =1.274262+0.001905u+0.000003u^{2}+0.0000002nu$$

**Figure 7:**
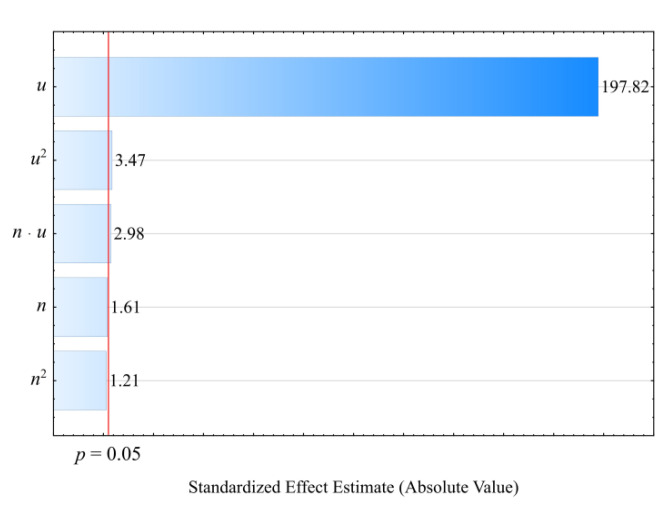
Pareto plots of the standardized effects of empirical full model density *ρ*, the vertical line in the plot corresponds to the arbitrarily chosen level of significance (*p* = 0.05).

**Figure 8:**
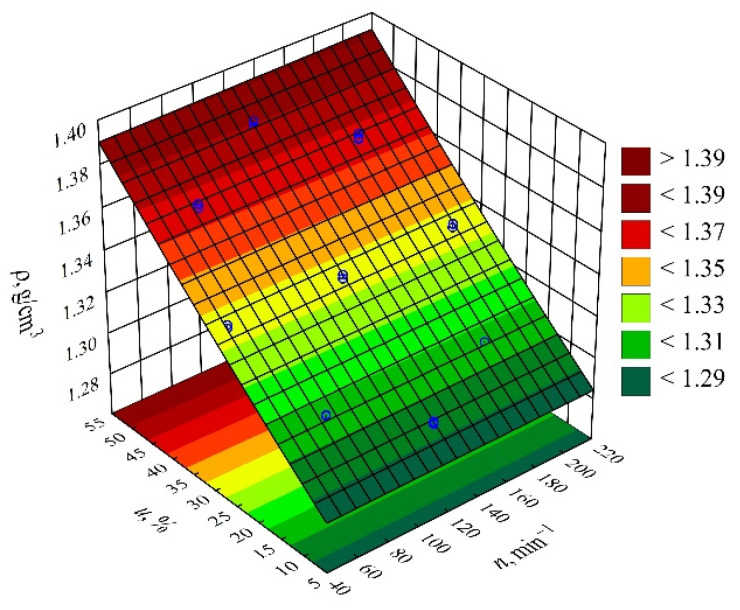
Response surface plot for the density *ρ* versus wheat bran content *u* and screw speed *n*.

It has been observed that the wheat bran content *u* introduced into the composition has the strongest effect on the density of the obtained mouldings (linear and quadratic terms of the equation—Figure 7). Also statistically significant was the interaction between bran content *u* and extruder screw speed *n* during the production of the composites under study, but its influence is relatively very small. The results of the statistical analysis of the adopted model are presented in Table 6. The linear term in the model equation has the greatest influence on the density. Increasing the bran content, the density of which is *ρ* = 1.5347 ± 0.0084 g/cm^3^, causes an increase in the density of the mouldings obtained from the polymeric composition (Figure 8). The highest increase in the density of the mouldings, 0.0861 g/cm^3^ (7%), was obtained by increasing the wheat bran content *u* in the composition from 10 to 50% (DOE layouts 7 and 8). The density of the samples made of PBS alone was significantly lower and amounted on average to *ρ* = 1.275 ± 0.0002 g/cm^3^, whereas the density of the filler was *ρ* = 1.5347 ± 0.0084 g/cm^3^. It should be noted that the density of the studied injection mouldings is in all cases clearly higher (from 0.0491 g/cm^3^ to as much as 0.1927 g/cm^3^) than the density of the pellets from which they were made. The relevant results of the density tests of the produced composition, in the form of pellets and microscopic pictures of its structure, were presented in a previous paper [66]. Reprocessing of the composition, this time by injection moulding, resulted in a decrease in the amount of pores present in the pellets as a result of the release of water vapour from the moisture contained in the bran and possibly partly from the products of the thermal decomposition of the composite components. During extrusion, there was a drastic reduction in pressure from 4–9 MPa to atmospheric pressure [66], which stimulated the formation of pores. During injection moulding, the material was pressed into the mould at 120 MPa and cooled at a gradient packing pressure of 120–80 MPa. This resulted in more packed material and prevented pore growth. The effect of pressure on the material packing was also confirmed in previous work with *p-v-T* T tests at 20 MPa and 110 MPa [66].

#### 3.1.3. Chemical Structure

**Figure 9:**
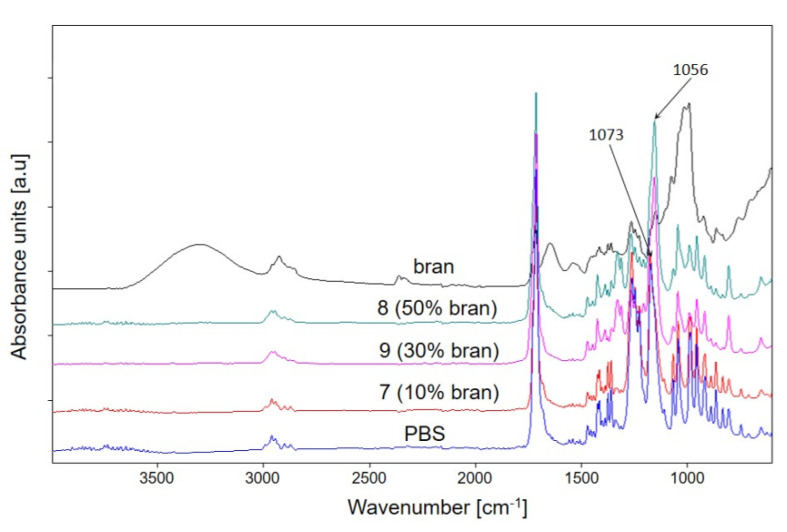
ATR-FTIR spectra of PBS, bran, and their composites 7, 8, 9 obtained with different bran content.

The chemical structure of PBS and its biocomposites with wheat bran was confirmed using FTIR analysis. Characteristic absorption bands originating from the vibrations of C=O carbonyl groups (1714 cm^−1^) and ester bonds -C-O-C and -O-(C=O) at 1262 cm^−1^, 1173 cm^−1^, and 1044 cm^−1^ are observed on PBS spectrum. The structure of wheat bran is mainly composed of polysaccharides (including cellulose, hemicellulose, and lignin), phenolic and lipid compounds, and proteins, as confirmed by the FTIR spectrum [66,91]. The spectra of PBS composites with bran (Figure 9) show absorption bands originating from functional groups that build both components. Figure 9 presents example FTIR spectra of the composites obtained at the same screw speed but with increasing bran content from 10% through 30% to 50%. An increase in the intensity of the absorption bands originating from the bran structure is observed along with an increase in the bran content in the composites. Particularly clear is the change in the maximum absorption band present on the PBS spectrum at 1173 cm^−1^ to 1156 cm^−1^ for composites 8 and 9, containing 50% and 30% bran, respectively. The shift of this band originating from the -C-O-C- vibration indicates the presence of a non-covalent interaction between PBS and the bran chemical components, which has also been previously reported [65,92]. It can be expected that this may be an interaction of the nature of hydrogen bonding, most likely between the -OH groups of the polysaccharides and lignin and the C=O groups in the polyester, but no shift in the absorption band of the carbonyl group was observed.

### 3.2. Thermal and Thermomechanical Properties

#### 3.2.1. DSC

**Figure 10:**
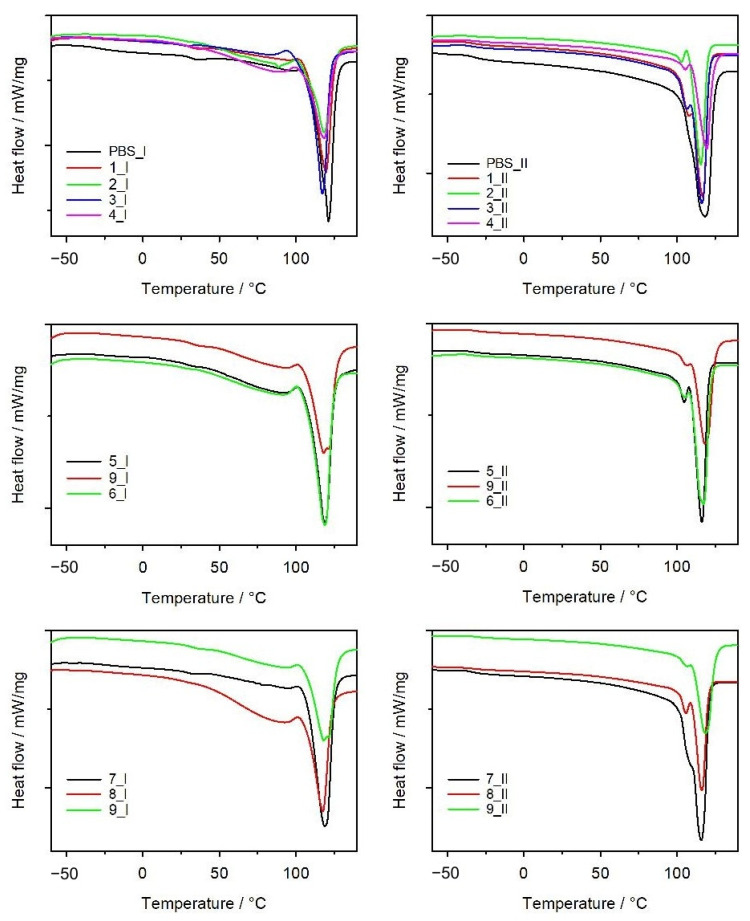
DSC thermograms of first (I) and second (II) heating scans of neat PBS and its composites with bran (exo up).

The DSC tests were performed in an inert gas atmosphere. The tests were performed in cycles: heating (I), cooling, and then heating (II). Table 7 presents the thermal parameters of the mouldings and their crystallinity degrees, determined on the basis of the DSC thermograms presented in Figure 10. The glass transition temperature (*T_g_*) of PBS determined from the first heating cycle is about 5 °C higher than that of the composites. This difference almost disappears when *T_g_* is read from the thermograms from the second heating cycle, and the secondary chain relaxation of PBS occurs at around −32 °C. The DSC thermograms show distinct endothermic peaks, which are due to the melting of the crystalline phase of the PBS present in the composites. The maximum of these endothermic transformations occurs at approx. 120 °C. However, the *T_m_* of neat PBS is slightly higher than that of the composites, and in general, the *T_m_* values determined from the first heating cycle are higher than those determined from the second heating cycle. Apart from the high PBS melting point, the DSC thermograms of the composites from the second heating cycle also show lower endothermic peaks with the maximum value at approx. 105 °C, while the curves from the first heating cycle show a broad peak extending from approx. 80 °C. The presence of this broad peak in the first heating cycle may be a result of the evaporation of a small amount of water absorbed within the structure of the composites, the presence of which was also confirmed by TG tests. The presence of a smaller endothermic peak on the thermograms from the second heating cycle has already been reported in our previous work [66], in which we presented DSC thermograms made for the pellets from which the injection mouldings under discussion were obtained. The course of the thermograms for the mouldings is different than that for the initial pellets for each composite, irrespective of the content of bran and screw speed; a clear first endothermic peak is present. If the mouldings with different bran content obtained with the same *n*, (1, 2 or 3, 4 or 7–9), are compared, a greater separation of endothermic peaks can be observed with the increasing bran content. As postulated earlier, the first endothermic peak is probably due to the melting of the less perfect PBS crystalline phase, which in this case was formed during the injection-moulding process. The values of the degree of crystallinity of the composite mouldings calculated from the first and second heating cycles are significantly different. Taking into account the fact that absorbed water is present in the structure of the composites, it can be concluded that the melting points of the crystalline phase and water desorption could overlap, which influenced the Δ*H_m_* value, as well as the values of the crystallinity degree. Therefore, when considering the effect of the bran content on the degree of crystallinity of the composites, we refer to the values determined from the second heating cycle. In the case of the mouldings, a dependence of *X_c_* on the content of bran is observed, which is analogous to the pellets. Neat PBS shows the highest crystallinity (60.5%) and increasing the content of bran in the composites decreases the *X_c_* values (DOE layouts 1, 2 or 3, 4 or 7–9). In contrast to the previously described pellets, the screw speed at which the output pellets were obtained did not affect the degree of crystallinity of the mouldings obtained in the injection-moulding process.

#### 3.2.2. Thermal Stability

**Figure 11:**
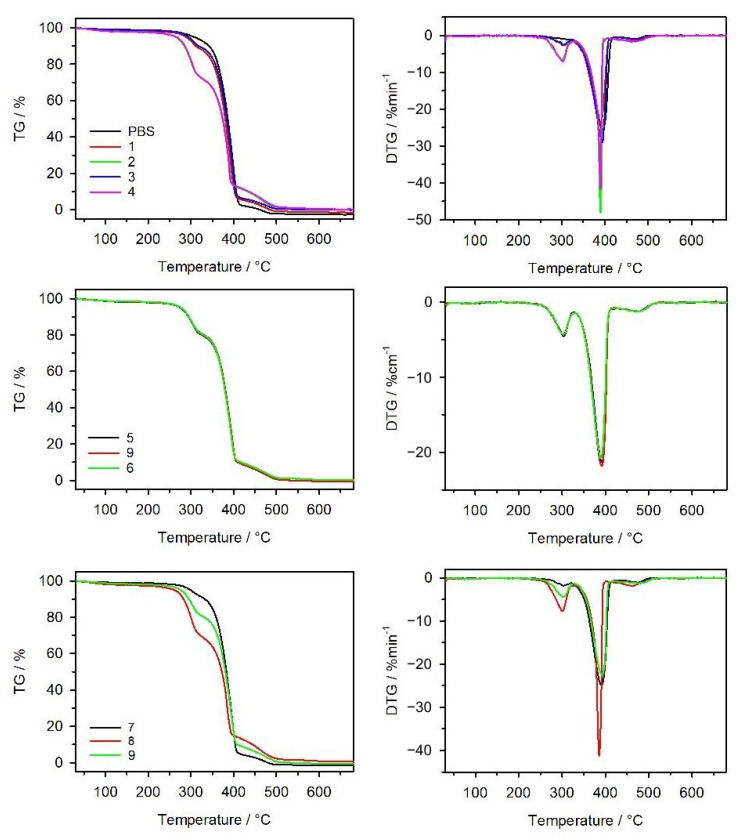
TG and DTG curves of PBS and its biocomposites with bran.

On the basis of a thermogravimetric analysis carried out in a synthetic air atmosphere, the thermal stability and thermal decomposition course of the obtained mouldings were determined. The TG curves presented in Figure 11 show a small mass loss, not exceeding 5%, related to the desorption of water present in the composite structure. The temperature at which 5% of the sample decomposed (*T*_5%_) was determined as the temperature of the onset of the mass loss of the samples. As can be seen from the data in Table 8, the *T*_5%_ value is the highest for neat PBS and decreases markedly with the increasing bran content in the composite structure. The lowest stability (261 °C) is shown by material 8, containing 50% bran. Comparing the thermal stability of the mouldings and the pellets, discussed in previous work, from which the mouldings were obtained, it can be stated that the injection moulding process did not affect the changes in the course of the thermal decomposition of the composites. The decomposition of neat PBS proceeds in a two-stage process; in the first stage, the hydrolysis of ester bonds occurs in parallel with the oxidation processes, whereas the second stage involves the final oxidation of the resulting deposit [66]. The decomposition of the composites proceeds in three stages; besides the mass loss associated with the decomposition of PBS, an additional first stage at about 303 °C, associated with the oxidative decomposition of the bran, is observed. The former mass loss is proportional to the bran content in the composites, which is also demonstrated by the TG and DTG curves of composites 1, 2 and 3, 4 and 7–9. An increase in the bran content causes a decrease in the thermal stability of the composites, which manifests itself in the values *T*_5%_ and *T*_50%_. Moreover, the *R_m_* values for the composites and PBS, indicating the residual mass of the sample after the TG analysis, point that they decompose completely at 800 °C. Coming back to the comparison of the thermal stability of the discussed mouldings with that of the initial pellets, it is evident that in the case of mouldings 5, 9, and 6, the *n* parameter has no influence on the *T*_5%_ values. During the injection-moulding process, the initial pellets were heated above the value *T_m_*, the internal structure of the composition was reorganized, and the effect of the conditions of the pellet preparation on the thermal stability of the moulded parts disappeared.

#### 3.2.3. Heat Deflection Temperature

The determined relation describing the variation of the heat deflection temperature HDT was presented by means of a polynomial (Equation (7)):

(7) HDT=90.24372+0.07126u 

**Figure 12:**
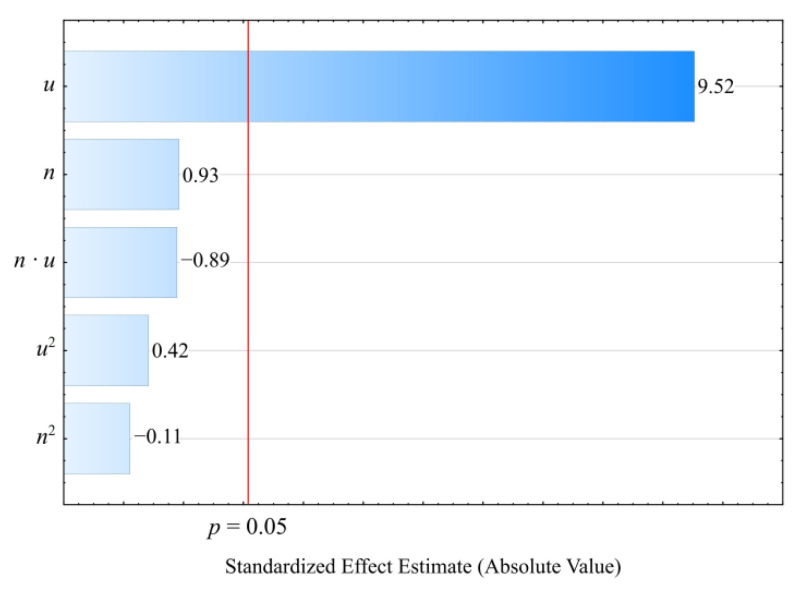
Pareto plots of the standardized effects of empirical full model of heat deflection temperature HDT, the vertical line in the plot corresponds to the arbitrarily chosen level of significance charts of the standardized effects for the empirical model *P* of the polymer blend pressure; the vertical line in the plot corresponds to the arbitrarily chosen level of significance (*p* = 0.05).

**Figure 13:**
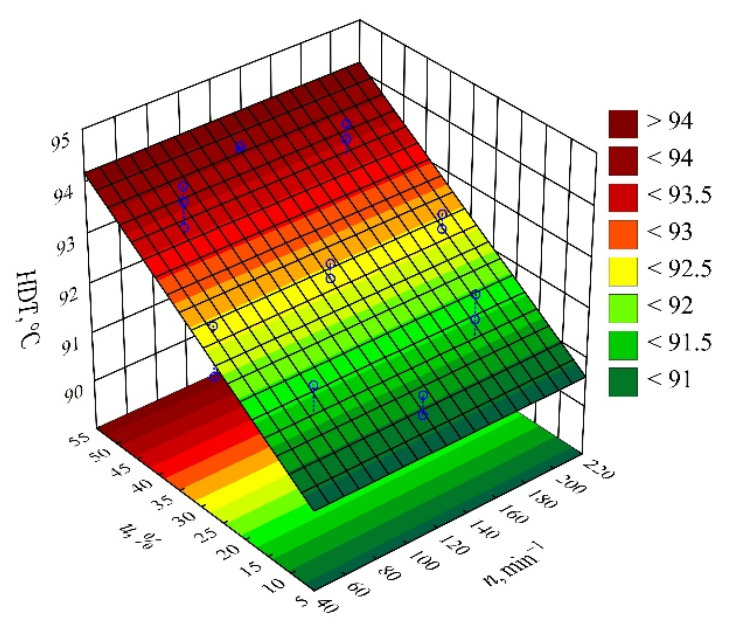
Response surface plot for the heat deflection temperature HDT versus wheat bran content *u* and screw speed *n*.

The results of the statistical analysis of the adopted *HDT* model are presented in Table 9. Similarly, as in the case of the other studied quantities, a significant linear effect on the values of the heat deflection temperature *HDT* of the compositions studied is exerted by the wheat bran content *u (*Figure 12). Increasing bran content causes a significant increase in *HDT* in comparison with the samples of PBS alone, for which the determined *HDT* was significantly lower and amounted on average to HDT = 88.2 ± 0.4 °C (Figure 13). The highest increase in *HDT* during the tests, i.e., 2.4 °C (3%), was obtained by increasing the wheat bran content *u* in the composition from 10 to 50% (DOE layouts 7 and 8). There was no significant effect on the *HDT* of the applied extruder screw speed *n* during the production of the polymer composite.

The increase in the HDT with the bran content is mainly due to the stiffening effect of the material. The presence of a dispersed, fine-grained natural filler in the PBS structure provides a mechanical barrier to the mobility of the macromolecules, improving the stiffness of the biocomposite and slowing down the deformation process. Therefore, it is necessary to reach temperatures in the higher range in order to obtain the preset bending deflection. Many authors obtain similar results, where the HDT value for the PBS matrix composites increases by several to over a dozen degrees relative to the unfilled PBS [43,76,93,94,95]. The literature also includes papers demonstrating the significant influence of interactions at the interfacial boundary of the PBS/natural filler, indicating the beneficial effect of compatibilizers on the HDT values [23,94]. Therefore, the non-covalent interactions between PBS and WB shown earlier in FTIR tests may also have a beneficial effect on the HDT values obtained for the PBS/WB composites under study.

#### 3.2.4. Vicat Softening Temperature

Based on the obtained measurement results, an empirical model describing the Vicat softening temperature VST (Equation (8)) was determined in the form of a polynomial:

(8) VST=109.7676−0.0307u

**Figure 14:**
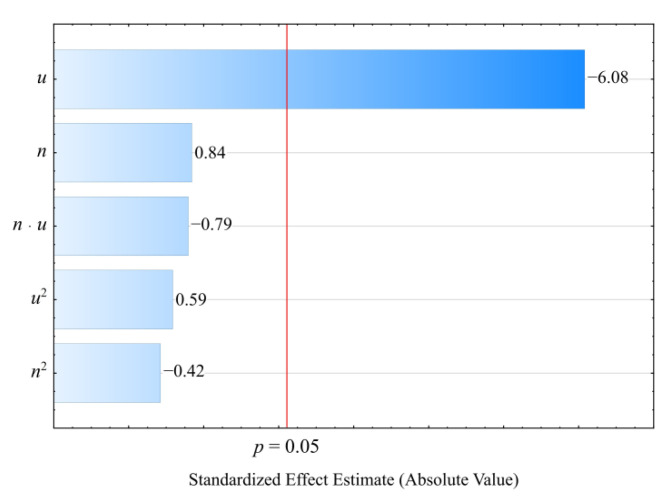
Pareto plots of the standardized effects of empirical full model Vicat softening temperature VST, the vertical line in the plot corresponds to the arbitrarily chosen level of significance (*p* = 0.05).

**Figure 15:**
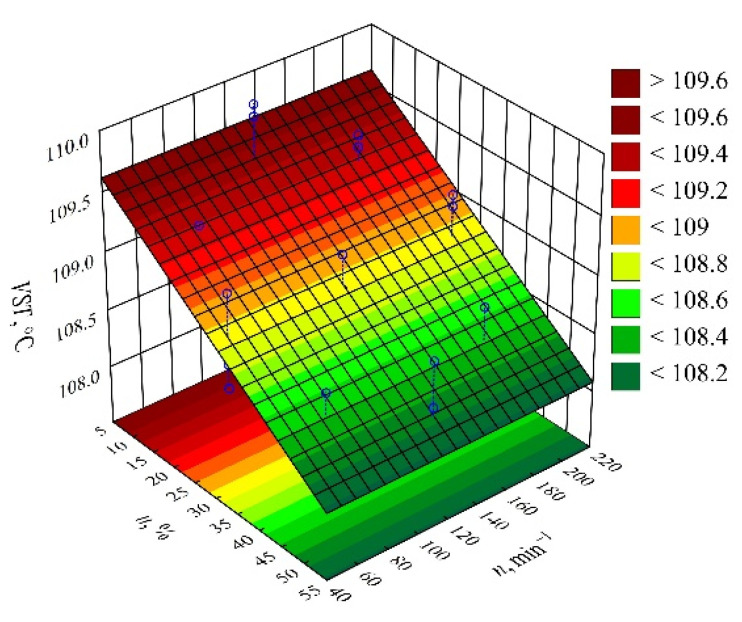
Response surface plot for the Vicat softening temperature VST versus wheat bran content *u* and screw speed *n*.

Statistical analysis of the effects of the studied variable factors on the *VST* showed a statistically significant effect of only the mass content of bran *u* (Figure 14). The results of the statistical analysis of the adopted model are presented in Table 10. In contrast to the HDT, as the bran content of the composition increases, the value of the Vicat softening temperature *VST* decreases in a linear fashion (Figure 15). In the case of the samples made of PBS alone, the Vicat softening temperature was higher and amounted on average to *VST* = 111.1 ± 0.2 °C. The observed changes, although statistically significant, are much smaller compared to those observed for the HDT. The largest observed decrease in the Vicat softening temperature *VST*, as a result of increasing the bran content in the composition from 10 to 50% (DOE layouts 7 and 8), was only 1.2 °C (change by 1%).

The VST is a parameter that indicates the beginning of the transformation of a polymeric material into a molten state. Thus, the obtained VST results for the studied compositions are in agreement with the DSC results (Table 7), based on which, the melting point of the PBS/WB biocomposites was observed to be lower than that of the unfilled PBS. The bran content also lowers melting enthalpy, meaning less energy is required to initiate the melting of the PBS/WB composite. Taking into account the *T_m_* results from the DSC tests, it can be concluded that for the PBS/WB composites the temperature range of the elastoplastic state has shifted slightly towards lower temperatures [96].

### 3.3. Mechanical Properties

#### 3.3.1. Hardness

The result of the performed modelling of hardness *H* of the injection mouldings made of the compositions under study is an empirical model in the form of a polynomial (Equation (9)):

(9) $$H=49.32992+0.44949u-0.00334u^{2}$$

**Figure 16:**
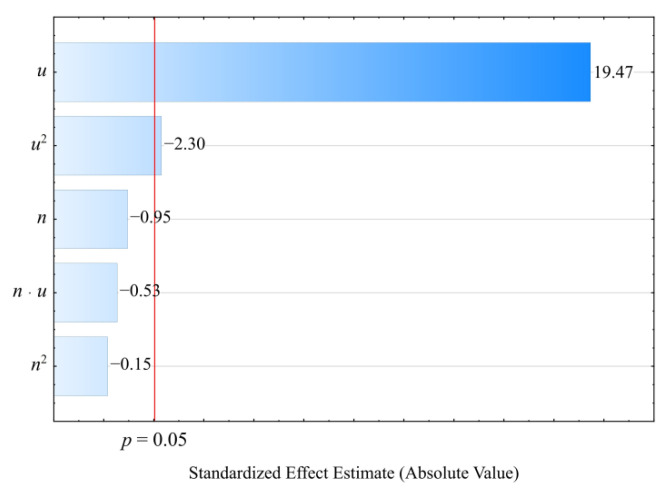
Pareto charts of the standardized effects for the empirical full model *H*; the vertical line in the plot corresponds to the arbitrarily chosen level of significance (*p* = 0.05).

**Figure 17:**
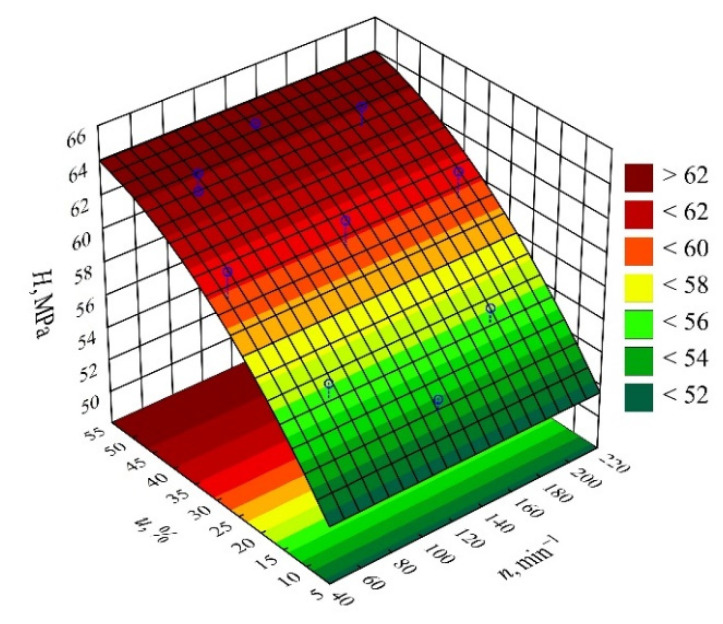
Response surface graph for the hardness *H* versus wheat bran content *u* and screw speed *n*.

The results of the statistical analysis of the adopted model are presented in Table 11. It was observed that the mass content of bran *u* introduced into the composite had a significant effect on the hardness of the obtained mouldings (Figure 16). The greatest influence is exerted by the linear term in the model equation, but the quadratic term is also statistically significant. However, its influence on hardness is many times smaller, and it has a negative effect. Increasing the bran content causes an increase in the hardness of the mouldings (Figure 17). The highest increase in hardness *H*, i.e., 10.3 MPa (19%), was obtained by increasing the wheat bran content *u* in the composition from 10 to 50% (DOE layouts 7 and 8). The statistical analysis, however, did not show any significant effect on the hardness of the mouldings of the extruder screw speed *n* applied during the production of the polymeric composition or the interaction between the bran content and the extruder screw speed. The hardness of the samples produced for comparison from PBS alone, without bran addition, was lower and averaged *H* = 49.55 ± 1.0 MPa.

The hardness of the composites containing powder fillers depends on many factors, which include the mechanical and physical properties of the filler itself (stiffness, hardness, and fineness) but also on the uniformity of the filler distribution in the polymeric matrix and on the quality of the interfacial interactions. A slight increase in the hardness of the PBS matrix composites with the increasing content of the powdered natural fillers is a typical result reported by many authors [97,98]. However, only the adequate mixing and compatibilization between the natural filler and the PBS ensure an effective force transfer and a significant increase in hardness [99]. Concurrently, the use of, e.g., maleinized or epoxidized vegetable oils leads to the disruption of the interfacial interactions and a lower hardness relative to the composites without added oils [25,67].

#### 3.3.2. Impact Strength

On the basis of the obtained measurement results, an empirical model was determined in the form of a polynomial describing the relation of the impact strength and the examined variable factors (Equation (10)):

(10) $$Impact\;strength=67.88949-2.21174u+0.02094u^{2}$$

**Figure 18:**
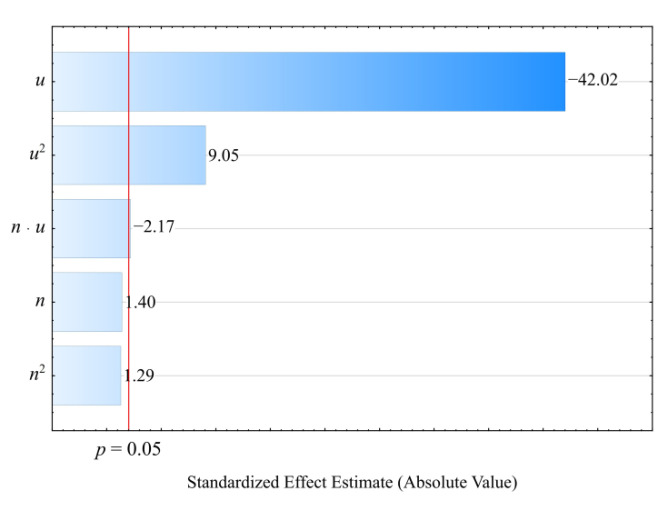
Pareto plots of the standardized effects of empirical full model impact strength; the vertical line in the plot corresponds to the arbitrarily chosen level of significance (*p* = 0.05).

**Figure 19:**
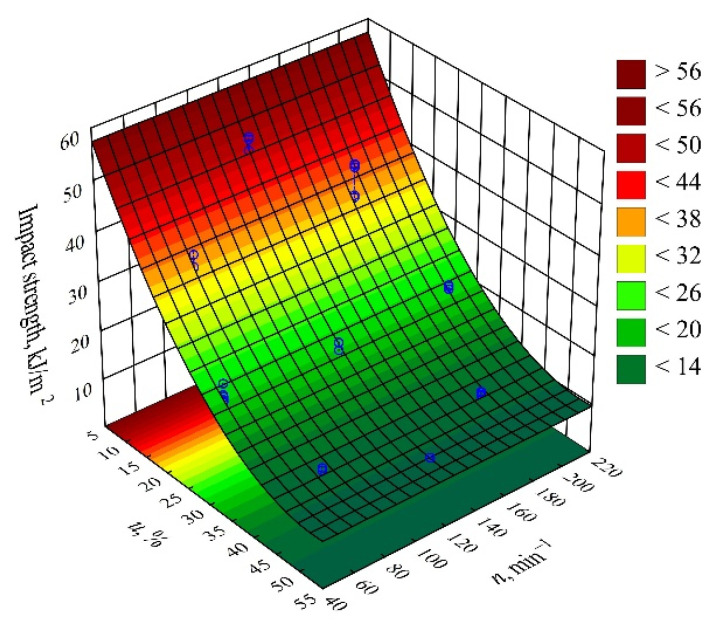
Response surface plot for the impact strength versus wheat bran content *u* and screw speed *n*.

The statistical analysis of the effects of the studied variable factors on *impact strength* showed a statistically significant effect of only the mass content of bran *u* (Figure 18). The results of the statistical analysis presented in Table 12 demonstrated the significance of the linear and quadratic terms in the adopted model. The *impact strength* values decrease sharply with the increase in wheat bran content *u* (Figure 19). As a result of a maximal increase in the wheat bran content *u* in the composite from 10 to 50% (DOE layouts 7 and 8), the value of the *impact strength* decreased by as much as 40.6 kJ/m^2^ (82%). The unfilled PBS samples were the only ones that did not fracture under the test parameters used.

The nature of the fracture of the PBS/WB biocomposite samples changed with their content. For the two lowest WB contents (10% and 16%), the formation of a partial spall was observed in the middle part of the sample. No spalling was observed for the other contents, but the roughness of the resulting fracture increased along with the increasing filler content. The unfilled PBS samples were the only ones that did not fracture under the test parameters used. Taking into account the glass transition temperature (Table 7) and the VST values (Table 1) of the tested materials, it can be concluded that at room temperature, at which the impact test was conducted, the materials remain in an elastic state. For this reason, the unfilled PBS did not crack. At low bran contents, there occurs an initial accumulation of impact energy in the form of elastic deformation, followed by the initiation of a rapid crack of a brittle nature with spalling due to stress concentration on a random material defect, such as microcracks at the interfacial boundary. The energy is mostly used to initiate the crack. The samples with filler content from 30 to 50% have a significantly lower impact strength values due to numerous material defects, which are potential places for crack initiation. The fracture is brittle in nature and the energy is mainly utilized for crack propagation; so, the crack resistance decreases drastically with filler content [100,101]. Deterioration of the impact strength with the increasing natural filler content is a typical phenomenon for PBS-based biocomposites [17,21,36,67,102].

#### 3.3.3. Tensile Strength

The determined relation describing the variation of tensile strength was presented by means of a polynomial (Equation (11)):

(11) $$\sigma =37.94986-0.55714u+0.00144u^{2}$$

**Figure 20:**
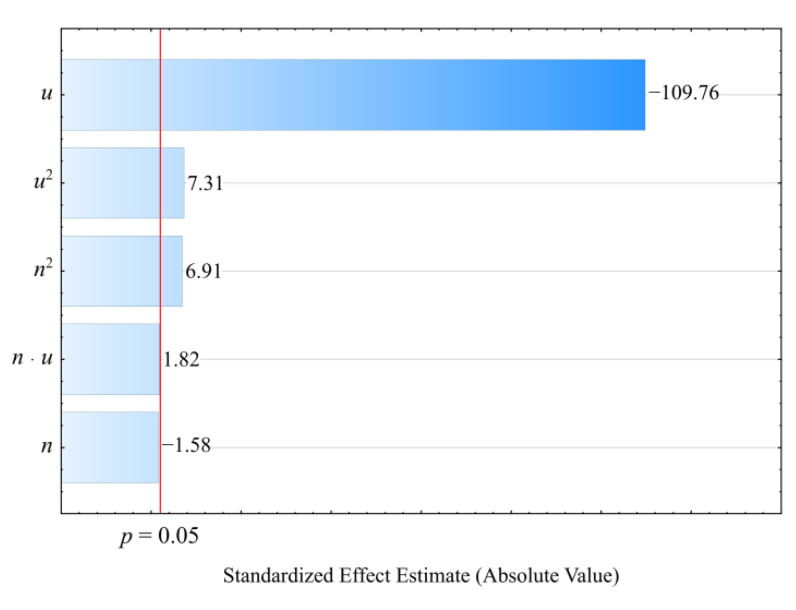
Pareto plots of the standardized effects of empirical full model tensile strength *σ*; the vertical line in the plot corresponds to the arbitrarily chosen level of significance (*p* = 0.05).

**Figure 21:**
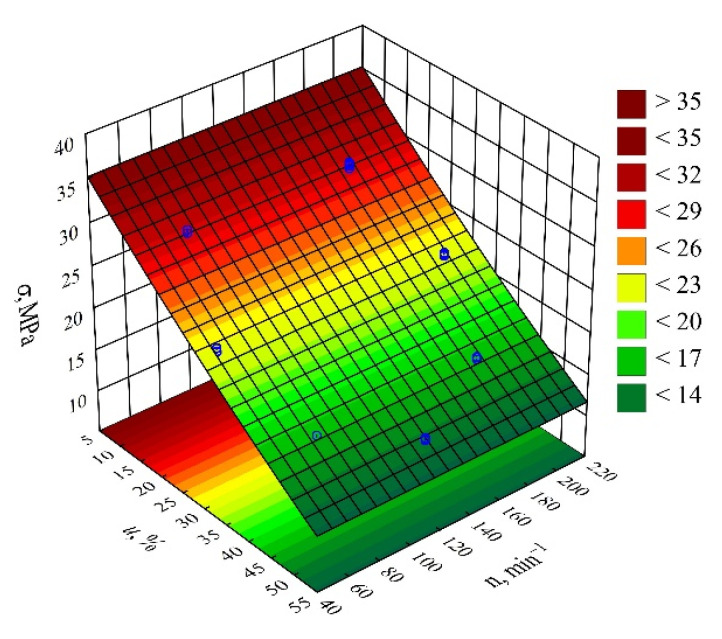
Response surface plot for the tensile strength *σ* versus wheat bran content *u* and screw speed *n*.

The initial analysis of the full model (Figure 20) indicated that the effect of both the wheat bran content *u* and the screw speed *n* was significant. However, the applied backward stepwise regression method of model building eventually demonstrated that, for this quantity, only the mass content of bran *u* (the linear and quadratic term) introduced into the composition has a significant effect on its values. The results of the statistical analysis of the adopted model *σ* are presented in Table 13. Moreover, in this case, increasing the bran content in the composition causes a significant decrease in the tensile strength (Figure 21). This is confirmed by the significantly higher tensile strength of the samples made of PBS alone, which was on average *σ* = 40.68 ± 0.52 MPa. During the tests, following the maximum increase in wheat bran content *u* in the composition from 10 to 50% (DOE layouts 7 and 8), the greatest decrease in tensile strength *σ* of 18.4 MPa (58%) was recorded. 

Tensile strength is strongly dependent on the quality of interactions at the matrix/filler interfaces and the quality of filler distribution in the matrix. Weak interaction forces cannot effectively transfer stresses between the filler grains and the polymer matrix, leading to the formation of microcracks and discontinuities. The lack of compatibilizer and the low strength of the bran itself leads to cavitation, i.e., the formation and enlargement of voids, which are the places where cracks initiate. A detailed description of the cavitation effect can be found in the work of Kim et al. [103]. As the WB content increases, so does the number of potential material defects that may become points of failure initiation during tension, especially for the hydrophilic WB and PBS matrix with a moderate affinity for water [21,43]. The description of the effect consisting in the decrease in tensile strength of the biocomposites based on the PBS matrix with the increase in natural filler content can be found in many works of other authors [21,36,37,38,40,41,42,43].

Moreover, during the processing PBS, unlike WB, is subject to the effect of processing shrinkage. This leads to a shrinkage of the plastic on the filler grains, exposing them to compressive stresses, while the matrix itself is then subjected to tensile stresses. The filler particles inside the PBS matrix consequently become stress concentration points, resulting in reduced tensile strength [104,105].

#### 3.3.4. Young’s Modulus

The result of the performed modelling of Young’s modulus *E* of the injection mouldings made of the compositions under study is an empirical model in the form of a polynomial (Equation (12)):

(12) E=616.7247+20.0558u

**Figure 22:**
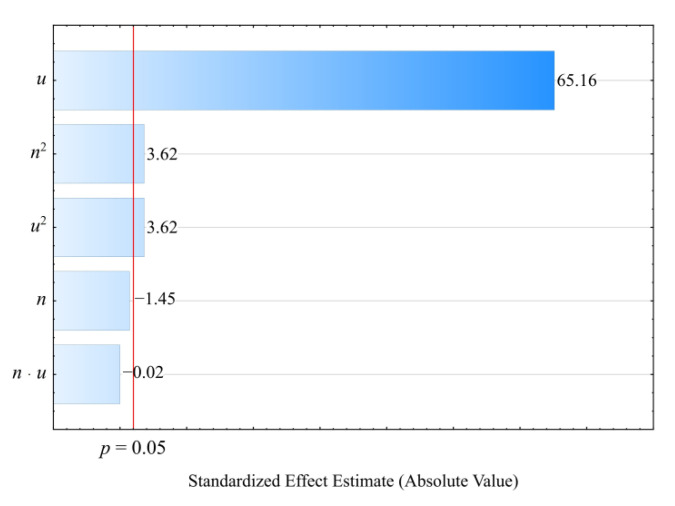
Pareto plots of the standardized effects of empirical full model Young’s modulus *E*; the vertical line in the plot corresponds to the arbitrarily chosen level of significance (*p* = 0.05).

**Figure 23:**
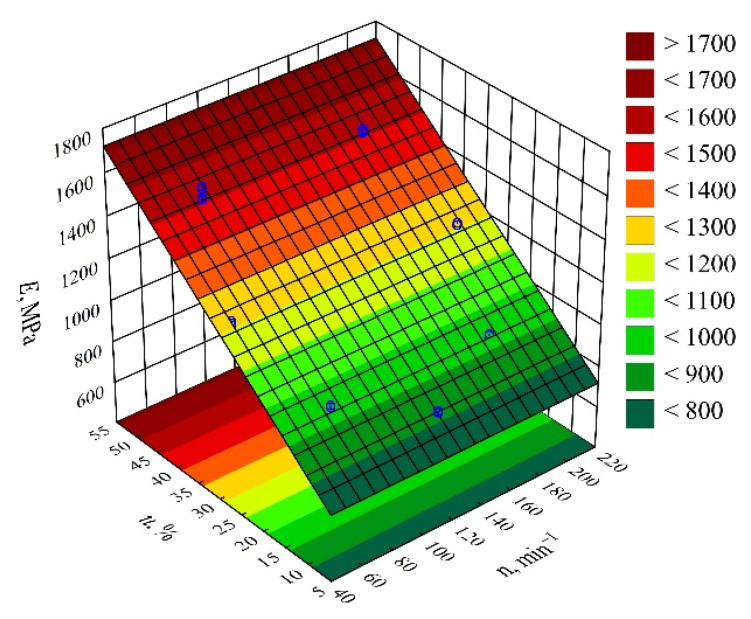
Response surface plot for the Young’s modulus *E* versus wheat bran content *u* and screw speed *n*.

Once again, the preliminary analysis of the full model (Figure 22) indicated that the influence of both variable factors under study was significant. The results of the modelling performed (Table 14), as the most suitable model, indicated the linear dependence of taking into account only the effect of wheat bran content *u*. The highest increase in Young’s modulus of the mouldings, i.e., 754 MPa (94%), was obtained by increasing the mass content of bran *u* in the composition from 10 to 50% (DOE layouts 7 and 8) (Figure 23). Young’s modulus of the samples made from the PBS alone was significantly lower and averaged *E* = 729 ± 8 MPa.

As mentioned earlier when describing the HDT results, the introduction of a powder filler into the polymer matrix results in an increase in stiffness by limiting the mobility of the macromolecules with the presence of a dispersed phase. The increase in stiffness is manifested by lower deformability, deterioration of the elastic and plastic properties, and an increase in brittleness, which clearly affects all the parameters related to the deformation of the samples. This is, of course, a common phenomenon occurring in polymer composites containing filler in powder form, either of natural or mineral origin [37,38,41,42,43,76].

#### 3.3.5. Elongation at Break

On the basis of the obtained measurement results, an empirical model was determined in the form of a polynomial describing the relation of elongation at break *ε* and the examined variable factors (Equation (13)):

(13) ε=29.42612−0.42370u

**Figure 24:**
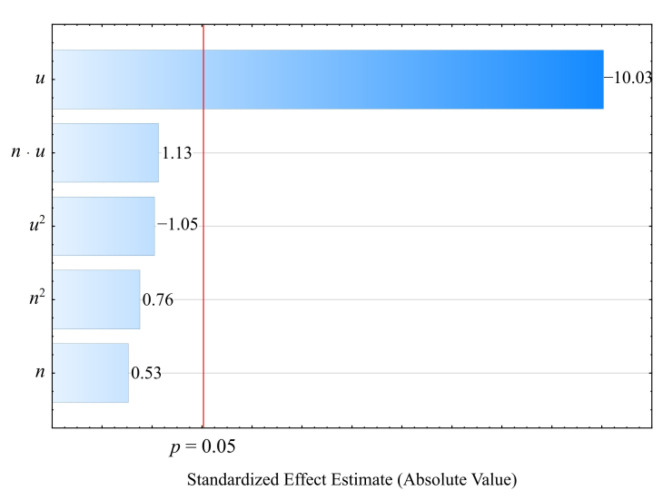
Pareto plots of the standardized effects of empirical full-model elongation at break *ε*; the vertical line in the plot corresponds to the arbitrarily chosen level of significance (*p* = 0.05).

**Figure 25:**
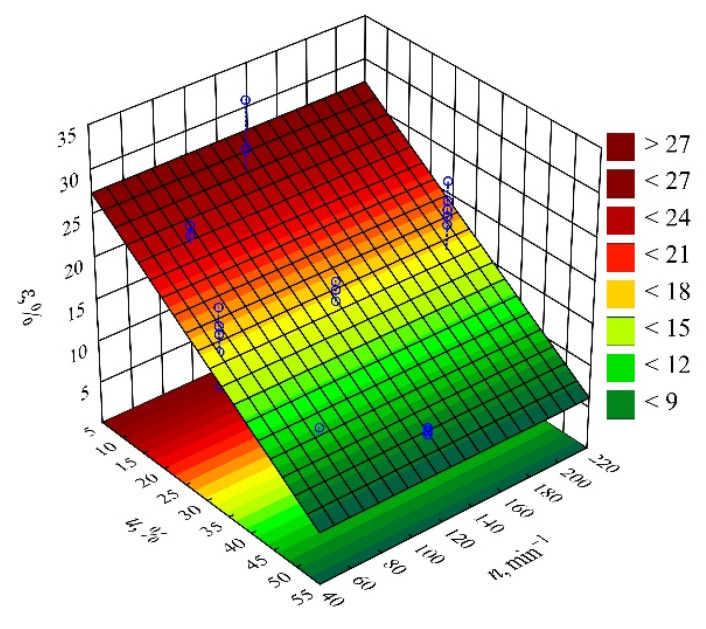
Response surface plot for the elongation at break *ε* versus wheat bran content *u* and screw speed *n*.

The statistical analysis of the effects of the studied variable factors on elongation at break showed a statistically significant effect of only the mass content of bran *u* (Figure 24). The results of the statistical analysis presented in Table 15 demonstrated the significance of the linear and quadratic terms in the adopted model of elongation at break. As the bran content *u* increases, the values of elongation at break *ε* decrease in a linear fashion (Figure 25). As a result of a maximal increase in the mass content of bran *u* in the composite from 10 to 50% (DOE layouts 7 and 8), the value of elongation at break *ε* decreased by as much as 18.4% (69% of the initial value). Samples made from the PBS alone had a significantly higher elongation at break *ε* = 218.6 ± 14.6%, and a ductile fracture with a very long neck.

The obtained course of change in elongation at break is related to the aforementioned increase in the stiffness of the composition and, at the same time, its brittleness, which is manifested by a significant decrease in deformability. Analogous courses of changes in the maximum deformation of the PBS with the increasing natural filler content can be observed in other works and for other fillers [37,41,42,43,76]. The nature of the obtained fractures also changed from ductile to brittle, similarly to the case with impact strength. Even the addition of 10% WB resulted in a reduction in deformation by almost 200%, but the fracture was ductile, and numerous longitudinal pore-like structures could be clearly observed on the surface of the neck as a result of the cavitation effect. Each such structure represents a potential point of crack initiation. At a bran content of 16%, there was only a residual neck. For 30% and 44% bran content, the neck did not occur, and plastic deformation was manifested in the form of light discolouration on the measurement part of the tested samples. On the other hand, at the content of 50%, no plastic deformation indicators visible to the naked eye were observed, and a brittle fracture with high roughness was obtained.

## 4. Conclusions

Analysing the presented work as a whole, it can be stated with certainty that there is no significant influence of the extruder screw speed during the production of pellets on all the properties of the injection-moulded parts made of those pellets. Thus, for the sake of efficiency and economy of production, high screw speed values are preferred during the extrusion of the composite pellets. As presented in a previous paper [66], this has a positive effect on the flow rate of the extrudate and minimizes the energy consumption due to the autothermal effect, while not exerting a negative effect on the properties of the finished injection-moulded products. Concurrently, a very significant effect of the filler content on the properties investigated was found.

A beneficial effect of WB on the processing shrinkage value was observed. As the filler content increases, the shrinkage values in all three directions decrease. Lower shrinkage values facilitate mould designing and help maintain the dimensional stability of the moulded parts. Moreover, the studied injection mouldings obtained higher density values in comparison with the pellets from which they were made. This is due to the high injection pressure and cooling under packing pressure.

Chemical structure tests showed the presence of structures and compounds typical for PBS and wheat bran. The possibility of non-covalent interactions between the matrix and the filler was also found.

The presence of WB in the structure of PBS affects its thermal properties. The DSC results demonstrated a significant effect of the presence of bran on the degree of crystallinity and the crystallization temperature of the PBS. The degree of crystallinity decreases relative to the unfilled PBS. This may affect the mechanical and thermal properties of the biocomposite to some extent. The melting point of the tested compositions is slightly lower than that for neat PBS. An analogous relationship was obtained for the VST. The crystallization temperature during cooling is also lower than for PBS alone. The HDT increased along with the increase in WB content, in spite of a general decrease in thermal stability shown in the TG tests.

As far as the mechanical properties are concerned, an increase in hardness and stiffness with the increasing WB content was demonstrated. The maximum tensile strength and impact strength deteriorated drastically, which is as expected and typical for PBS matrix composites containing powdered fillers of natural origin. In spite of the observed deterioration of some of the mechanical properties, their values still remain at a satisfactory level and can meet the design requirements of many objects of everyday use. The high filling degree of WB allows for an effective reduction in the cost of manufacturing PBS components, potentially contributing to the industrial popularity of this biodegradable material.

## Figures and Tables

**Table 1:** Experimental design and experimental test results—mean values and standard deviation—part I.

Experimental Design Layout	*n*, min^−1^	*u*, %	*S_L_*, %	*S_T_*, %	*S_P_*, %	*ρ*, g/cm^3^	*HDT*, °C	*VST*, °C
1	72	15.9	1.07 ± 0.01	1.83 ± 0.05	2.11 ± 0.02	1.3064 ± 0.0010	91.1 ± 0.8	109.1 ± 0.3
2	72	44.1	0.61 ± 0.01	1.37 ± 0.06	1.11 ± 0.01	1.3652 ± 0.0010	93.7 ± 0.4	108.4 ± 0.2
3	178	15.9	1.07 ± 0.01	1.88 ± 0.06	2.12 ± 0.02	1.3054 ± 0.0007	91.5 ± 0.8	109.3 ± 0.3
4	178	44.1	0.61 ± 0.01	1.37 ± 0.06	1.03 ± 0.03	1.3668 ± 0.0012	93.6 ± 0.5	108.3 ± 0.5
5	50	30.0	0.91 ± 0.01	1.66 ± 0.03	1.99 ± 0.02	1.3352 ± 0.0008	92.0 ± 0.5	108.7 ± 0.4
6	200	30.0	0.89 ± 0.01	1.58 ± 0.06	1.90 ± 0.03	1.3361 ± 0.0008	92.4 ± 0.4	108.9 ± 0.4
7	125	10.0	1.23 ± 0.01	2.03 ± 0.05	2.13 ± 0.02	1.2938 ± 0.0005	91.2 ± 0.2	109.7 ± 0.3
8	125	50.0	0.57 ± 0.01	1.11 ± 0.04	0.93 ± 0.02	1.3798 ± 0.0014	93.6 ± 0.5	108.5 ± 0.3
9 (C)	125	30.0	0.90 ± 0.01	1.58 ± 0.06	1.94 ± 0.02	1.3349 ± 0.0010	92.3 ± 0.4	108.8 ± 0.3

**Table 2:** Experimental design and experimental test results—mean values and standard deviation—part II.

Experimental Design Layout	*n*, min^−1^	*u*, %	*H*, MPa	*Impact* *Strength*, kJ/m^2^	*σ*, MPa	*E*, MPa	*ε*, %
1	72	15.9	55.9 ± 1.09	35.17 ± 1.53	30.20 ± 0.20	953 ± 5	22.1 ± 2.0
2	72	44.1	63.0 ± 0.47	11.70 ± 0.24	16.18 ± 0.30	1554 ± 27	9.1 ± 1.4
3	178	15.9	55.6 ± 1.19	38.96 ± 3.44	29.90 ± 0.27	929 ± 8	18.1 ± 1.7
4	178	44.1	62.2 ± 0.92	11.45 ± 0.34	16.58 ± 0.11	1530 ± 7	9.3 ± 1.1
5	50	30.0	59.8 ± 1.40	20.91 ± 1.43	23.00 ± 0.23	1208 ± 13	18.0 ± 3.4
6	200	30.0	59.7 ± 1.19	21.00 ± 2.09	22.34 ± 0.15	1206 ± 5	21.8 ± 1.9
7	125	10.0	53.3 ± 1.16	49.49 ± 1.48	31.96 ± 0.05	830 ± 3	26.6 ± 7.4
8	125	50.0	63.6 ± 0.47	8.88 ± 0.76	13.54 ± 0.13	1584 ± 9	8.1 ± 0.9
9 (C)	125	30.0	59.8 ± 1.40	19.44 ± 1.37	21.54 ± 0.11	1172 ± 8	16.9 ± 1.5

**Table 3:** Model of longitudinal shrinkage S*_L_*—ANOVA table, R^2^ = 0.98; R_adj_^2^ = 0.98.

Source of Variation	SS	df	MS	F	*p*
*U*	2.123473	1	2.123473	2285.24	0.00000
Error	0.039956	43	0.000929		
Total SS	2.163429	44			

SS—sum of squares, df—number of the degrees of freedom, MS—mean sum of squares, F—values of the test statistic, *p*—value of probability corresponding to the test statistic value.

**Table 4:** Model of transverse shrinkage S*_T_*—ANOVA table, R^2^ = 0.94; R_adj_^2^ = 0.94.

Source of Variation	SS	df	MS	F	*p*
*u*	3.259054	1	3.259054	655.59	0.00000
Error	0.213760	43	0.004971		
Total SS	3.472814	44			

SS—sum of squares, df—number of the degrees of freedom, MS—mean sum of squares, F—values of the test statistic, *p*—value of probability corresponding to the test statistic value.

**Table 5:** Model of perpendicular shrinkage S*_P_*—ANOVA table, R^2^ = 0.97; R_adj_^2^ = 0.96.

Source of Variation	SS	df	MS	F	*p*
*u*	8.997936	1	8.997936	1063.73	0.00000
*u* ^2^	1.160503	1	1.160503	137.19	0.00000
Error	0.355273	42	0.008459		
Total SS	10.513711	44			

SS—sum of squares, df—number of the degrees of freedom, MS—mean sum of squares, F—values of the test statistic, *p*—value of probability corresponding to the test statistic value.

**Table 6:** Model of density *ρ*—ANOVA table, R^2^ = 0.99; R_adj_^2^ = 0.99.

Source of Variation	SS	df	MS	F	*p*
*u*	0.036588	1	0.036588	37,269.04	0.000000
*u* ^2^	0.000011	1	0.000011	11.68	0.001441
*nu*	0.000008	1	0.000008	8.43	0.005907
Error	0.000040	41	0.000001		
Total SS	0.036648	44			

SS—sum of squares, df—number of the degrees of freedom, MS—mean sum of squares, F—values of the test statistic, *p*—value of probability corresponding to the test statistic value.

**Table 7:** Melting point (*T_m_*), crystallization (*T_c_*), and glass transition (*T_g_*) temperatures; the enthalpy of melting (Δ*H_m_*) and degree of crystallinity (*X_c_*) of PBS and its composites with bran, based on differential scanning calorimetry (DSC) thermograms.

Sample	Heating I				Cooling	Heating II			
	*T_g_* [°C]	*T_m_* [°C]	Δ*H_m_* [J/g]	*X_c_* [%]	*T_c_* [°C]	*T_g_* [°C]	*T_m_* [°C]	Δ*H_m_* [J/g]	*X_c_* [%]
PBS	−25.6	121.3	72.1	65.4	86.4	−31.7	118.5	66.7	60.5
1(16)	−32.5	119.6	58.0	62.6	85.3	−32.1	116.8	54.1	58.4
2(44)	−33.5	118.4	41.0	66.4	78.0	−33.8	115.5	35.4	57.3
3(16)	−30.2	117.3	67.5	72.9	84.3	−33.9	116.3	54.8	59.1
4(44)	−29.9	118.3	43.6	70.6	80.6	−32.0	118.5	34.4	55.7
5(30)	−31.3	118.9	51.1	66.2	82.2	−32.1	116.3	38.2	49.5
6(30)	−31.5	118.9	51.7	67.0	81.3	−32.4	117.2	39.4	51.0
7(10)	−33.3	118.8	63.3	63.8	86.4	−33.4	115.8	55.3	55.7
8(50)	−32.5	117.6	39.0	70.7	83.3	−32.5	116.6	30.6	54.5
9(30)	−31.4	118.0	41.9	54.3	79.6	−32.3	118.2	38.8	50.3

**Table 8:** Parameters characterizing the thermal stability of PBS, bran, and biocomposites, obtained based on thermogravimetry (TG) and derivative thermogravimetry (DTG) curves.

	*T*_5%_ [°C]	*T*_50%_ [°C]	*T_max_*_1_ [°C]	Δ *m*_1_ [%]	*T_max_*_2_ [°C]	Δ *m*_2_ [%]	*T_max_*_3_ [°C]	Δ *m*_3_ [%]	*R_m_* [%]
bran	201	303	296	68.0	-	-	459	29.7	2.3
PBS	307	386	-	-	395	97.9	463	2.0	0.1
1(16)	288	380	303	11.9	389	82.0	474	6.0	0.1
2(44)	264	375	301	27.8	389	59.1	459	12.9	0.2
3(16)	293	384	303	10.7	394	82.3	478	6.9	0.1
4(44)	266	374	303	27.9	390	58.7	462	13.2	0.2
5(30)	274	380	303	20.5	392	70.3	476	9.1	0.1
6(30)	276	379	303	20.1	390	70.0	476	9.7	0.2
7(10)	299	383	303	8.5	391	87.0	475	4.5	0.3
8(50)	261	372	300	31.5	386	53.5	462	14.7	0.3
9(30)	274	381	303	19.8	391	71.1	476	9.0	0.1

**Table 9:** Model of heat deflection temperature HDT—ANOVA table, R^2^ = 0.80; R_adj_^2^ = 0.79.

Source of Variation	SS	df	MS	F	*p*
*u*	24.36606	1	24.36606	98.33462	0.000000
Error	6.19468	25	0.24779	-	-
Total SS	30.56074	26	-	-	-

SS—sum of squares, df—number of the degrees of freedom, MS—mean sum of squares, F—values of the test statistic, *p*—value of probability corresponding to the test statistic value.

**Table 10:** Model of Vicat softening temperature VST—ANOVA table, R^2^ = 0.64; R_adj_^2^ = 0.62.

Source of Variation	SS	df	MS	F	*p*
*u*	4.140601	1	4.140601	42.56	0.000001
Error	2.334784	24	0.097283	-	-
Total SS	6.475385	25	-	-	-

SS—sum of squares, df—number of the degrees of freedom, MS—mean sum of squares, F—values of the test statistic, *p*—value of probability corresponding to the test statistic value.

**Table 11:** Model of hardness *H*—ANOVA table, R^2^ = 0.92; R_adj_^2^ = 0.91.

Source of Variation	SS	df	MS	F	*p*
*u*	456.4350	1	456.4350	424.7162	0.000000
*u* ^2^	9.5844	1	9.5844	8.9184	0.004921
Error	40.8379	38	1.0747		
Total SS	500.6960	40			

SS—sum of squares, df—number of the degrees of freedom, MS—mean sum of squares, F—values of the test statistic, *p*—value of probability corresponding to the test statistic value.

**Table 12:** Model of impact strength—ANOVA table, R^2^ = 0.98; R_adj_^2^ = 0.97.

Source of Variation	SS	df	MS	F	*p*
*u*	6223.385	1	6223.385	1574.945	0.000000
*u* ^2^	376.598	1	376.598	95.305	0.000000
Error	154.108	39	3.951		
Total SS	6468.503	41			

SS—sum of squares, df—number of the degrees of freedom, MS—mean sum of squares, F—values of the test statistic, *p*—value of probability corresponding to the test statistic value.

**Table 13:** Model of tensile strength—ANOVA table, R^2^ = 0.99; R_adj_^2^ = 0.99.

Source of Variation	SS	df	MS	F	*p*
*u*	1726.567	1	1726.567	5428.771	0.000000
*u* ^2^	2.027	1	2.027	6.375	0.015538
Error	13.040	41	0.318		
Total SS	1741.222	43			

SS—sum of squares, df—number of the degrees of freedom, MS—mean sum of squares, F—values of the test statistic, *p*—value of probability corresponding to the test statistic value.

**Table 14:** Model of Young’s modulus *E*—ANOVA table, R^2^ = 0.99; R_adj_^2^ = 0.99.

Source of Variation	SS	df	MS	F	*p*
*u*	3,216,923	1	3,216,923	3198.350	0.000000
Error	43,250	43	1006		
Total SS	3,260,173	44			

SS—sum of squares, df—number of the degrees of freedom, MS—mean sum of squares, F—values of the test statistic, *p*—value of probability corresponding to the test statistic value.

**Table 15:** Model of elongation at break *ε*—ANOVA table, R^2^ = 0.72; R_adj_^2^ = 0.72.

Source of Variation	SS	df	MS	F	*p*
*u*	1243.739	1	1243.739	104.1111	0.000000
Error	477.851	40	11.946		
Total SS	1721.590	41			

SS—sum of squares, df—number of the degrees of freedom, MS—mean sum of squares, F—values of the test statistic, *p*—value of probability corresponding to the test statistic value.

## Data Availability

The data presented in this study are available on request from the corresponding author.

## List of Abbreviations and Symbols

PBS	Poly(butylene succinate)
WB	Wheat bran
DOE	Design of experiment
FTIR	Fourier transform infrared (spectroscopy)
DSC	Differential scanning calorimetry
*X_c_*	Degree of crystallinity
Δ*H_m_*	Melting enthalpy
*T_m_*	Melting point
*T_c_*	Crystallization temperature
*T_g_*	Glass transition temperature
TG	Thermogravimetry
DTG	Derivative thermogravimetry
*T*_5%_, *T*_50%_	Temperature of 5% and 50% of mass loss
*T_max_*	Temperature of the maximum rate of mass loss
Δ*m*	Mass loss corresponding to *T_max_*
*R_m_*	Residual mass
*U*	Wheat bran content
*N*	Extruder screw speed
*HDT*	Heat deflection temperature
*VST*	Vicat softening temperature
*LCF*	Lignocellulosic filler
*α*	Star point distance in DOE
*S_L_*	Longitudinal shrinkage
*S_T_*	Transverse shrinkage
*S_P_*	Perpendicular shrinkage
*Ρ*	Density
*p-v-T*	Relationship between pressure *p*, specific volume *v* and temperature *T*
*H*	Hardness
*σ*	Tensile strength
*E*	Young’s modulus
*ε*	Elongation at break
